# Chinese Expert Consensus for the Application of rh-aFGF for Chronic Refractory Wounds

**DOI:** 10.3390/ph19020328

**Published:** 2026-02-17

**Authors:** Junli Zhou, Xuanru Zhu, Dongcheng Ye, Xiaobing Fu, Yuesheng Huang, Professional Committee of Wound Repair of Chinese Medical Doctor Association

**Affiliations:** 1 Department of Wound Repair, Burn and Plastic Surgery, Dongguan People’s Hospital, The Tenth Affiliated Hospital of Southern Medical University, Dongguan 523059, China; yedongcheng0769@sina.com; 2 Department of Plastic Surgery, The First Affiliated Hospital of Jinan University, Guangzhou 510632, China; xuanruzhu@163.com; 3 Research Center for Wound Repair and Tissue Regeneration, Medical Innovation Research Department, The PLA General Hospital, Beijing 100853, China; fuxiaobing@vip.sina.com; 4 Institute of Wound Repair and Regeneration Medicine, Technology School of Medicine, Southern University of Science, Shenzhen 518055, China

**Keywords:** fibroblast growth factor, recombinant human acidic fibroblast growth factor (rh-aFGF), chronic refractory wound, expert consensus

## Abstract

**Background and Objective:** Chronic refractory wounds not only persist and delay healing, causing physical and mental suffering to patients and reducing their quality of life but also impose a heavy burden on their families and society. Recombinant human acidic fibroblast growth factor (rh-aFGF), approved in China for decades, is a growth factor with multiple biological effects which can promote the healing of various wounds. It is crucial to develop an rh-aFGF expert consensus for standardizing the clinical application of aFGF and enhancing its clinical value. **Methods:** The literature related to rh-aFGF, including clinical trials, experimental research, and reviews, was collected and selected from PubMed, Web of Science, Medline, CNKI, and the Wangfang database. The expert recommendations were formed by a combination of clinical research quality, use of the Delphi questionnaire, and consensus reached during meetings involving experts. **Results:** A total of 12 consensus recommendations for clinical application of rh-aFGF on chronic refractory wounds (CRWs) were successfully formulated, of which seven strong, four moderate, and one weak recommendations were suitable for various clinical sets. The recommendations include specifications for duration of rh-aFGF treatment, dosage, types of wounds, and its combination with other products and dressings. **Conclusions:** rh-aFGF has vital clinical value for healing chronic wounds. This consensus provides clinicians with a reference to guide the application not only of rh-aFGF but also of other growth factor drugs with similar mechanisms for CRWs.

## 1. Introduction

The International Society of Regenerative Medicine and Wound Repair has defined chronic refractory wounds (CRWs) as wounds that fail to undergo a timely, normal, and orderly physiological repair process to achieve full structural and functional recovery despite standardized and systematic treatment [1,2,3,4,5]. Most scholars consider a wound to be a CRW if it remains unhealed or shows no tendency to heal after 4 weeks or more of standardized and comprehensive treatment [3]. CRWs caused by various reasons (including traumas and other diseases) persist, delay healing, and are difficult to treat, which not only severely affect patients’ work and quality of life but also imposes a heavy burden on their families and society. CRWs represent a major focus and challenge in current clinical research and treatment. CRWs include diabetic foot ulcers, pressure ulcers, vascular wounds, tophaceous wounds, malignant cutaneous wounds, chronic wounds from superficial tumors or abscesses, radiation ulcer wounds, iatrogenic wounds (delayed healing of surgical incisions), tuberculous wounds, chronic osteomyelitis-related wounds, wounds caused by special bacterial infections, chronic wounds resulting from traumas and burns in both peacetime and wartime, and scar or ulcer wounds. In China alone, more than 30,000 patients require hospitalization for such conditions each year [6]. Wound treatment includes surgical and non-surgical approaches. As an important nonsurgical technique for wounds, recombinant human acidic fibroblast growth factor (rh-aFGF) has been used for treating more than one million cases of various acute and chronic wounds in clinical practice for nearly 20 years, demonstrating favorable efficacy with no significant toxic effects or adverse reactions reported to date. However, since CRWs, such as diabetic ulcers and pressure ulcers, vary widely in etiology and presentation, the standardized application of rh-aFGF must be tailored for the special characteristics of individual wounds to achieve optimal therapeutic efficacy. To this end, the Wound Repair Committee of the Chinese Medical Doctor Association organized experts to propose these consensus recommendations on the application of rh-aFGF for chronic refractory wounds based on the principles of evidence-based medicine. These recommendations are intended to serve as references for relevant clinical practitioners.

aFGF belongs to the FGF family. To date, 22 members of this family have been identified and classified into seven subfamilies [7] which are involved in the regulation of endocrine functions, embryonic development, and injury repair [8]. rh-aFGF is an aFGF constructed by recombinant DNA technology. Studies have indicated that exogenous rh-aFGF exerts its biological effects by binding to fibroblast growth factor receptors (FGFRs) on target cells. This binding induces intracellular tyrosine phosphorylation of FGFRs, thereby activating the receptors. Heparin, as a key cofactor, facilitates the binding of rh-aFGF-FGFRs and FGFR dimerization to accelerate its activation [9]. Activated FGFRs further activate multiple downstream signaling pathways, including RAS-MAPK and PI3K-AKT pathways, which can induce the expression of nuclear transcription factors, upregulate cell cycle-related proteins, and promote cell division and proliferation [10]. Inflammatory responses play a dual role in wound healing. Moderate early inflammation helps eliminate pathogens and promotes chemotactic recruitment of reparative cells, facilitating wound healing; however, persistent and excessive inflammation is a primary cause of delayed healing in chronic wounds. Studies have shown that rh-aFGF regulates inflammation and promotes tissue repair through multiple mechanisms. Specifically, rh-aFGF can activate the RAS-MEK-ERK pathway and inhibit the activation of STAT proteins to reduce the expression of inflammatory cytokines such as IL-6 [11]. By upregulating the expression of the transcription factor Nrf-2, rh-aFGF promotes the synthesis of peroxiredoxins, accelerates ROS metabolism, and alleviates oxidative stress and inflammation [12]. rh-aFGF can also regulate the polarization of macrophages from the proinflammatory M1 phenotype to the anti-inflammatory M2 phenotype, accelerating wound repair [13]. In the late stage of wound healing, rh-aFGF further promotes re-epithelialization, induces fibroblast apoptosis, and reduces scar formation [14]. Compared with basic fibroblast growth factor (bFGF), rh-aFGF can bind to all fibroblast growth factor (FGF) receptors and exhibits stronger activity [1,15].

As an endogenous trace active substance in the human body, aFGF is difficult to extract from body fluids or tissues, posing a challenge for research and development of aFGF. Chinese scientists have constructed genetically engineered bacteria expressing rh-aFGF using recombinant DNA technology. Under the control of a modern, high-standard GMP quality management system, advanced bioengineering techniques are employed to achieve large-scale production of rh-aFGF, making clinical application of aFGF possible. In 2006, rh-aFGF was approved by the NMPA of China as the first aFGF used in clinical practice worldwide [16]. Currently, rh-aFGF is still in the research phase overseas and has not been approved for clinical use. However, other related growth factor products, including EGF, bFGF, and PDGF, are available [17]. Their indications, dosages, administration frequencies, and countries in which they are approved are summarized in Table 1.

Based on the specific characteristics of various CRWs and the principles of evidence-based medicine, this consensus aims to standardize the application of rh-aFGF for CRWs with the goal of optimizing its therapeutic efficacy and preventing misuse. The target population of this consensus includes patients with superficial CRWs. This consensus may also serve as a reference for the treatment of patients with acute wounds. This consensus provides references for clinical practitioners in medical institutions managing patients with CRWs, such as clinicians in wound repair, burn and plastic surgery, and orthopedics departments.

## 2. Results and Discussion

### 2.1. General Information

A total of 23 publications related to rh-aFGF clinical use, including 18 clinical trials of CRWs and five guidelines, were selected for considerations of consensus. Detailed information regarding these publications and the quality of their evidence (according to the GRADE System) is listed in Table 2.

**Figure 1:**
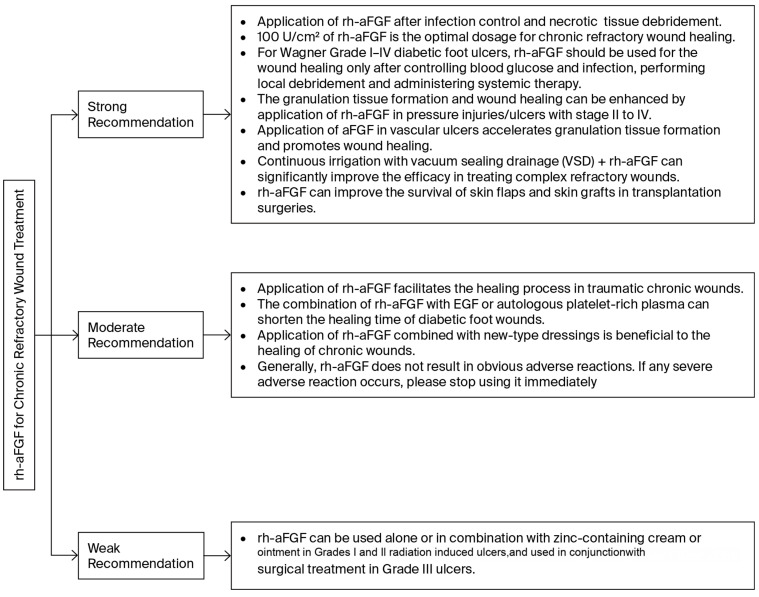
Schematic consensus recommendations for application of rh-aFGF on chronic refractory wounds.

According to clinical situations of CRWs, the consensus expert group determined 12 recommendations for rh-aFGF application; these are shown in Figure 1. Results of the details of clinical questions and recommendations are listed and discussed in the following sections.

**Figure 2:**
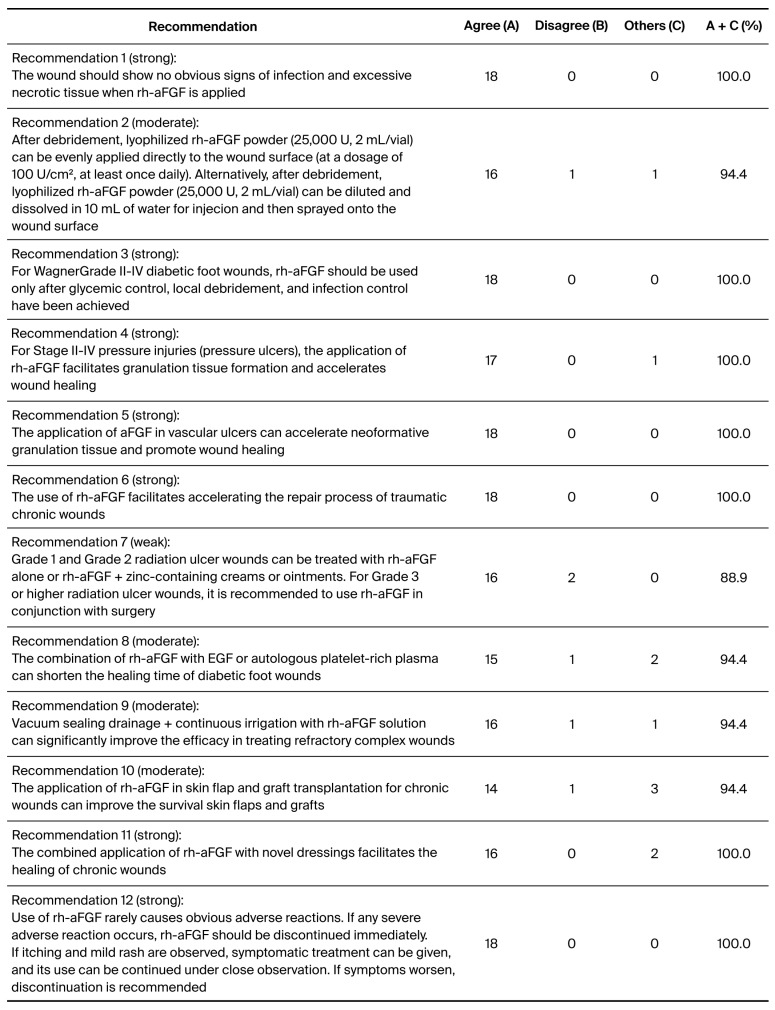
Results of the Delphi questionnaire survey.

Results of the recommendation questionnaire survey are shown in Figure 2. A total of 12 consensus recommendations for clinical application of rh-aFGF on chronic refractory wounds (CRWs) were successfully formulated. Of these, seven were strong (≥95%), four were moderate (<94%, ≥90%), and one was a weak (≤89%, ≥85%) recommendation suitable for various clinical sets.

### 2.2. Clinical Question 1: Timing of rh-aFGF Application

#### Recommendation 1 (Strong): The Wound Should Show No Obvious Signs of Infection and Excessive Necrotic Tissue When rh-aFGF Is Applied

Evidence and rationale: Clinical studies have shown that patients with chronic wounds (e.g., Stages III and IV pressure ulcers) are observed to exhibit significantly elevated levels and persistently high expression of inflammatory cytokines (IL-1β, IL-6, TNF-α) and apoptotic factor (Caspase-3) compared to those with acute wounds. This leads to suppression of the FGF synthesis and its receptor expression, as well as fibroblast apoptosis [36]. Other clinical studies have also demonstrated that bacteria and exudates present in wounds contain matrix metalloproteinases that can degrade FGF [37], which impairs the efficacy of topically applied aFGF. Furthermore, elastase produced by Pseudomonas aeruginosa in wounds can degrade plasma proteins and extracellular products of human skin and fibroblasts, including various antiproteases, complement C3, kininogen, fibroblast proteins, and proteoglycan decorin, thereby inhibiting fibroblast proliferation [38]. Therefore, thorough wound debridement is required before application of rh-aFGF to remove obvious necrotic tissue and effectively control severe wound infection. Topical application of rh-aFGF is not recommended immediately for wounds with severe periwound inflammatory reactions and significant exudation [17].

### 2.3. Clinical Question 2: Usage and Dosage of rh-aFGF

#### Recommendation 2 (Strong): After Debridement, Lyophilized rh-aFGF Powder (25,000 U, 2 mL/Vial) Can Be Evenly Applied Directly to the Wound Surface (at a Dosage of 100 U/cm^2^ and at Least Once Daily). Alternatively, After Debridement, Lyophilized rh-aFGF Powder (25,000 U, 2 mL/Vial) Can Be Diluted and Dissolved in 10 mL of Water for Injection and Then Sprayed onto the Wound Surface

Evidence and rationale: Studies have shown that rh-aFGF is stable in the slightly acidic environment of wounds, which is more conducive to rh-aFGF exerting its biological activity. Topical application is safe, as the amount of the drug absorbed into the blood circulation through wounds is extremely low, and it primarily exerts its pharmacological effects locally at the wound site without significant accumulation observed, showing no obvious impact on liver or kidney function and no accumulative toxic reactions [18]. Therefore, rh-aFGF is mainly administered topically. For a deep wound with heavy exudation, lyophilized rh-aFGF powder can be directly dissolved in the exudate, resulting in a higher local drug concentration and stronger permeability. It can penetrate through eschar and purulent secretions to reach the surface of granulation tissue, directly stimulating wound healing. When the lyophilized powder is evenly sprayed, it achieves a larger contact area with the wound without surface tension. By promoting cell division and proliferation and inhibiting exudation, direct application of lyophilized rh-aFGF powder yields better results [18,19]. For superficial wounds or those with minimal exudation, lyophilized rh-aFGF powder (25,000 U, 2 mL/vial) can be diluted and dissolved in 10 mL of water for injection and then directly sprayed onto the wounds after debridement. In a clinical study involving 139 cases of diabetic wounds, patients were randomly divided into the rh-aFGF group (*n* = 104) and the rh-bFGF group (*n* = 35) in a 3:1 ratio. The periwound skin was disinfected with 75% alcohol. After simple debridement, each wound was covered with a single layer of sterile gauze, followed by the 1000 U/mL rh-aFGF solution (prepared by dissolving a 25,000 U/vial of lyophilized rh-aFGF powder in sterile normal saline with a 25-fold dilution) applied dropwise at a dosage of 100 U/cm^2^. The required amount of the drug-containing solution was calculated based on the wound area and dripped onto the gauze until it was fully saturated. The wound was then dressed in petrolatum paraffin gauze and sterile dry gauze, respectively, once daily for 6 weeks. The overall healing rate in the rh-aFGF group reached 80%, demonstrating superior efficacy compared with the rh-bFGF group [20].

More attention should be paid to the application of rh-aFGF for CRWs:

(1) Different types of wounds should undergo wound bed preparation according to conventional methods. The recommended dosage of this product is currently 100 U/cm^2^. Either lyophilized powder or solution can be used for spraying—spray is recommended for dry wounds, while powder is suggested for wounds with excessive exudate. The wound can be dressed in Vaseline gauze, sterile gauze, or other wound dressings. This product can be used until the wound heals; if the wound does not heal within 2 months, it is recommended to switch to other products.
(2) Before applying this product, if protein denaturants such as iodine, ethanol, or hydrogen peroxide are used for wound bed preparation, the wound must be repeatedly rinsed with normal saline before using rh-aFGF to avoid affecting the product’s activity.

### 2.4. Clinical Question 3: Application of rh-aFGF in CRWs

#### 2.4.1. Recommendation 3 (Strong): For Wagner Grades II–IV Diabetic Foot Wounds, rh-aFGF Should Be Used Only After Glycemic Control, Local Debridement, and Infection Control Have Been Achieved

Evidence and rationale: The results of the clinical comparative study on the application of rh-aFGF and rh-bFGF for diabetic wounds showed that among 139 patients with diabetic wounds larger than 2 cm^2^ that had remained unhealed for more than 8 weeks, 104 and 35 cases were administered with rh-aFGF and rh-bFGF, respectively, both at a dosage of 100 U/cm^2^ once daily, to observe the complete healing rate and response rate. It was found that after 35 days of application, both the complete healing rate and the response rate in the rh-aFGF group were better than those in the rh-bFGF group by over 30%. These findings indicate that rh-aFGF is more effective than rh-bFGF in promoting the healing of diabetic wounds [20].

Promoting granulation tissue growth is a prerequisite for the repair of deep wounds [39]. In small deep wounds, granulation tissue growth accelerates the re-epithelialization process, while in large deep wounds, it can provide a wound bed for subsequent surgical intervention. Studies have confirmed that rh-aFGF can significantly promote granulation tissue growth and accelerate epithelialization [40,41], making it a preferable option for the repair of deep wounds. A randomized multicenter clinical study including 97 patients with Wagner Grades III–IV diabetic foot ulcers demonstrated that the application of rh-aFGF, in addition to systemic treatments such as glycemic control and active anti-infective therapy, significantly shortened wound healing time and reduced scar formation. Notably, the group receiving direct topical application of lyophilized powder combined with spray (n = 32) exhibited significantly better outcomes in terms of wound exudation, cumulative wound healing rate at 20 days, and complete healing time compared with the spray-only group (n = 34) and the group receiving moist exposed burn ointment (n = 31) [19]. Furthermore, a meta-analysis of 17 randomized controlled trials (RCTs) involving 934 patients with diabetic foot ulcers (DFUs) demonstrated [42] that the rh-aFGF groups achieved significantly higher efficacy than the control groups, with a markedly shorter wound healing time and no significant adverse reactions reported, indicating good safety. Pharmacoeconomic study results showed [43] that the rh-aFGF regimen is superior to the conventional treatment (normal saline) in terms of clinical efficacy and cost-effectiveness for treating patients with DFUs.

#### 2.4.2. Recommendation 4 (Strong): For Stages II–IV Pressure Injuries (Pressure Ulcers), the Application of rh-aFGF Facilitates Granulation Tissue Formation and Accelerates Wound Healing

Evidence and rationale: Pressure ulcers are among the most common chronic refractory wounds. According to the International Pressure Ulcer Guideline, Stage II pressure injuries present as intact or open serous blisters or may also appear as pink wounds [44]. In a clinical study, 30 patients diagnosed with Stage II pressure injuries were randomly divided into the rh-aFGF group and the foam dressing group, with 15 patients in each group. The rh-aFGF group had a total of 28 wounds (11 at the sacrococcygeal regions, 10 at the heels, and seven at the hips), while the control group had 26 wounds (eight at the sacrococcygeal regions, 13 at the heels, and five at the hips). The rh-aFGF powder was evenly applied to the wounds until healing. The results showed that the wound healing time in the rh-aFGF group was 6 days earlier than that in the foam dressing group (14 ± 4 days vs. 20 ± 6 days) [21], significantly shortening the hospitalization duration and reducing the economic burden on patients. A randomized, multicenter clinical study enrolled 75 patients with pressure ulcers occurring at bony prominences such as the sacra and ischial tuberosities. After debridement, they were treated topically with lyophilized rh-aFGF powder at a dose of 100 U/cm^2^. The control group received topical moist exposed burn ointment once daily for 20 consecutive days. The results revealed that compared with the control group, the cumulative healing rate in the rh-aFGF group at 20 days increased by 37%, and the complete healing time was approximately 5 days earlier, indicating that direct topical application of lyophilized rh-aFGF powder was more effective than moist exposed burn ointment in promoting healing. Additional studies have found that rh-aFGF can significantly accelerate the healing of Stages III and IV pressure injuries (pressure ulcers), promote the functional recovery of patients, and reduce their financial burden [19]. Under conditions of combined systemic nutritional support and dynamic assessment of wound progression, for Stage III and above pressure ulcers requiring surgical intervention (such as skin grafting/flap repair), local application of rh-aFGF after thorough debridement and infection control can promote fibroblast migration, microvascular network reconstruction, and collagen deposition in the wound and accelerate the formation of granulation tissue bed to fill the wound defect. This provides ideal wound conditions for subsequent surgical timing, shortens the preoperative preparation time, and improves the surgical success rate [45].

#### 2.4.3. Recommendation 5 (Strong): The Application of aFGF in Vascular Ulcers Can Accelerate Neo-Formative Granulation Tissue and Promote Wound Healing

Evidence and rationale: Vascular ulcers are a relatively common type of chronic refractory ulcer disease, including arterial ulcers, venous ulcers, and mixed ulcers of the lower extremities, etc. With the acceleration of population aging and the annual increasing incidence of chronic diseases, the incidence of vascular ulcers of lower extremities is also on the rise. Vascular ulcers of the lower extremities often do not heal normally. Their delayed healing is the result of underlying arterial, venous, and/or lymphatic diseases, and they may recur repeatedly [46]. Venous ulcers are often caused by varicose veins of the calf, venous inflammation, and poor local blood circulation in the lower legs, with an incidence of 1.5% [47]. A clinical study involving 105 patients with varicose ulcers received anti-infection and debridement for their wounds in addition to vascular treatment. Their wounds were treated with lyophilized rh-aFGF powder evenly applied topically, covered with sterile gauze, and then sprayed with rh-aFGF solution at a dose of 200 U/cm^2^ once daily for one month or until complete healing. The results demonstrated that patients receiving topical application of rh-aFGF combined with spraying of rh-aFGF solution had less wound exudation, better granulation growth, and accelerated wound healing at least 5 days earlier than those treated with spraying alone in the control group [19].

#### 2.4.4. Recommendation 6 (Moderate): The Use of rh-aFGF Facilitates Accelerating the Repair Process of Traumatic Chronic Wounds

Evidence and rationale: Various traumas and burns may develop into chronic refractory wounds due to delayed or improper management in their early stages. Forty patients with traumatic chronic wounds of the lower extremity were enrolled in a study, including 23 cases of wound infections after pretibial traumas, eight cases of congestive ulcers due to venous injuries of lower legs, four cases of non-healing wounds after Achilles tendon surgery, two cases of soft tissue defects of the heels, and three cases of infections after dorsalis pedis traumas. The wound sizes ranged from 1.0 cm × 2.0 cm to 4 cm × 8.5 cm. After debridement, the wounds were directly covered with gauze soaked in rh-aFGF solution (prepared by dissolving 2 mL of lyophilized rh-aFGF powder in 10 mL of water for injection). For sinus tracts and subcutaneous cavities, gauze strips soaked in the solution were loosely packed into the cavities, with one end of the gauze exposed externally to serve as a drug-loaded dressing and drainage strip. The wounds were then gently dressed with sterile dressings, with a frequency of dressing change of at least once daily [18]. For sinus wounds or wounds with larger defects, the dosage could be appropriately increased. The overall response rate reached 97.50% (39/40), and the marked effective rate was 92.50% (33/40).

#### 2.4.5. Recommendation 7 (Weak): Grades I and II Radiation Ulcer Wounds Can Be Treated with rh-aFGF Alone or rh-aFGF + Zinc-Containing Creams or Ointments; For Grade III or Higher Radiation Ulcer Wounds, It Is Recommended to Use rh-aFGF in Conjunction with Surgery

Evidence and rationale: Chronic radiation ulcers have long been a clinical challenge due to their complex pathogenesis characterized by delayed onset, progression, long duration, and irreversibility. They often involve deep vital tissues such as bones, nerves, and large blood vessels, severely affecting patients’ quality of life [48]. According to the diagnostic criteria for severity grading of chronic radiation-induced skin injuries, they are classified into Grade I (chronic radiation dermatitis), Grade II (induration and edema), and Grade III (chronic radiation ulcers) [49]. Based on the Common Terminology Criteria for Adverse Events (CTCAE), chronic radiation skin ulcers can be graded into five levels [50]. The Expert Consensus on Diagnosis and Treatment of Radiation-induced Skin Injuries (2024) recommends that FGF, zinc-containing creams, or ointments can be used for small and superficial ulcers to promote wound healing once infection is basically controlled. For any large, deep, and persistent chronic radiation ulcer, surgical debridement and timely repair should be performed as early as possible to prevent serious complications once infection is basically controlled and the patient’s general condition permits. FGF may be applied locally to facilitate repair [48,49,51].

### 2.5. Clinical Question 4: Combined Application with Other Growth Factor-Containing Products

#### Recommendation 8 (Moderate): The Combination of rh-aFGF with EGF or Autologous Platelet-Rich Plasma Can Shorten the Healing Time of Diabetic Foot Wounds

Evidence and rationale: Multiple studies have demonstrated that the combined use of rh-aFGF with EGF can significantly promote the healing of diabetic foot wounds. In a study, 199 patients were divided into four groups: the EGF, aFGF, combination (EGF + rh-aFGF), and normal saline control groups. The results showed that the complete wound healing time in the combination group (36.31 ± 1.62 days) was 11 days shorter than that in the control group (47.52 ± 1.82 days), indicating a marked synergistic effect of rh-aFGF + EGF in promoting healing [22]. Another prospective study involving 90 patients with Wagner Grades II–IV diabetic foot ulcers revealed significant differences in wound healing times among the three groups after interventions. Group A (sustained VSD treatment) had a healing time of 56.88 ± 10.64 days, Group B (VSD + lavage with rh-aFGF solution) had a shortened healing time of 35.64 ± 10.78 days, and Group C (the treatment regime in Group B plus autologous platelet-rich gel) had an even further shortened healing time of 29.72 ± 9.23 days. This indicates that rh-aFGF combined with platelet-rich gel can significantly accelerate the healing of refractory wounds [23]. Another study included 50 patients with chronic wounds and compared the efficacy between the treatment group (25 cases receiving VSD + rh-aFGF + platelet-rich plasma) and the control group (25 cases receiving VSD + rh-aFGF). The results showed that the wound healing time (19.42 ± 2.14 days vs. 26.47 ± 2.98 days), the preparation for secondary repair, and the hospital stay in the treatment group were all significantly shorter than those in the control group. On day 7 and day 14 after treatment, the treatment group achieved better bacterial culture negative conversion rates (61.90% vs. 25.00%; 95.24% vs. 55.00%) and greater reductions in C-reactive protein (CRP) levels compared with the control group. This suggests that platelet-rich plasma combined with rh-aFGF and VSD can effectively improve bacterial clearance, alleviate inflammation, and shorten the treatment cycle [24]. Collectively, these studies have demonstrated that rh-aFGF alone or rh-aFGF combined with EGF, platelet-rich plasma, and VSD can significantly shorten the healing time of diabetic foot ulcers, improve bacterial clearance and inflammatory responses, and serve as an effective strategy for promoting the repair of diabetic foot wounds.

### 2.6. Clinical Question 5: Combined Use with Other Wound Treatment Techniques

#### 2.6.1. Recommendation 9 (Strong): VSD + Continuous Irrigation with rh-aFGF Solution Can Significantly Improve the Efficacy in Treating Refractory Complex Wounds

Evidence and rationale: for refractory wounds with exposed deep tissues, such as Wagner Grade III or higher diabetic foot ulcers, pressure ulcers, fractures, or chronic osteomyelitis with bone exposure, exogenous continuous supplementation of rh-aFGF combined with VSD technique has been proven to effectively improve the wound environment, remove exudate, alleviate inflammatory responses, and promote granulation tissue growth. The closed and moist environment created by VSD is more conducive to maintaining the physiological activity of rh-aFGF. Multiple randomized controlled clinical studies demonstrated that this combined regimen was better than the conventional irrigation with normal saline alone. The overall response rate of the combination therapy was significantly higher than that of irrigation with normal saline, with the wound healing rate improved by at least approximately 30% [25,26,27,28,29,30]. Compared with the control group, the combined therapy significantly reduced the wound cavity volume (by 15.2 mL, 15.9 ± 1.9 mL vs. 0.7 ± 0.1 mL) and greatly improved the wound healing rate (by 52.3%, 60.9 ± 4.6% vs. 8.6 ± 3.1%) [25]. A multicenter clinical study involving 196 patients showed that the combination group had a higher granulation tissue score (2.6 ± 1.5 vs. 1.9 ± 1.4), a shorter complete healing time by approximately 5 days (14.6 ± 5.2 days vs. 19.5 ± 7.9 days), a reduced length of hospital stay (13.4 ± 4.7 days vs. 16.4 ± 5.2 days), and a smaller residual wound area (15.4 ± 1.9 cm^2^ vs. 19.3 ± 2.1 cm^2^) [29]. After the patients with Wagner Grade III diabetic foot ulcers were treated with intermittent negative pressure + continuous irrigation with rh-aFGF solution for seven days, they were observed to achieve significantly improved wound infection indicators (white blood cells, erythrocyte sedimentation rate, CRP), better granulation growth (score 1.39 ± 0.66 vs. 0.89 ± 0.05 scores), and significantly reduced wound cavity volume (11.14 ± 1.14 mL vs. 3.88 ± 2.10 mL) compared with the control group receiving VSD + irrigation with normal saline [31].

For the patients with fractures complicated by chronic osteomyelitis, the regime of VSD + continuous irrigation with rh-aFGF solution demonstrated significant advantages in the third week. The improvement rate of wound cavity volume (75.72 ± 2.10% vs. 32.54 ± 0.99%) and the healing rate of exposed bone tissue (62.82 ± 2.48% vs. 14.51 ± 1.95%) in the combination group were significantly higher than those in the control group [28]. The VSD system was used to provide personalized treatments such as oxygen delivery and drug delivery to wounds by its built-in irrigation tube. For continuous irrigation with rh-aFGF solution, five vials of rh-aFGF were added to the irrigation solution (1000 mL of normal saline), with the concentration maintained at 100–200 U/mL for continuous irrigation and drainage. The treatment cycle typically involved a VSD change every 5–7 days, with a total drainage time of 7–10 days. This could be extended up to three weeks for chronic osteomyelitis wounds [26,29]. rh-aFGF (25,000 U/cm^2^ mL) was mixed with normal saline at a dose of 100 U/cm^2^ and sprayed onto the wounds once daily for a 3-week course (suitable for fractures complicated by chronic osteomyelitis) to achieve satisfactory results [28]. The therapy of VSD + continuous irrigation with rh-aFGF solution provides a better treatment option for complex refractory wounds, such as Wagner Grade III or higher diabetic foot ulcers, pressure ulcers, fractures with bone exposure, and chronic osteomyelitis with bone exposure, to effectively accelerate healing and improve prognosis by optimizing the wound microenvironment and continuously supplying key growth factors.

#### 2.6.2. Recommendation 10 (Strong): The Application of rh-aFGF in Skin Flap and Graft Transplantation for Chronic Wounds Can Improve the Survival of Skin Flaps and Grafts

Evidence and rationale: In a clinical study involving 42 patients who received sural neurovascular flap transplantation combined with rh-aFGF for repairing skin and soft tissue defect wounds of heels, it was found that the survival area of skin flaps in the rh-aFGF treatment group was significantly greater than that in the control group [(98.4 ± 12.8)% vs. (89.4 ± 13.7)%]; the complete healing times at recipient sites [(9.4 ± 2.4) d vs. (13.8 ± 4.6) d] and donor sites [(11.4 ± 4.2) d vs. (16.7 ± 6.8) d] in the rh-aFGF treatment group were significantly shorter than those in the control group. The edema, exudation, and dark purple flap scores in the treatment group were significantly lower than those in the control group. During a 30-day follow-up period, one case of infection occurred in the treatment group, with an incidence rate of 4.7%, while eight cases of infection occurred in the control group, with an infection rate of up to 28.5%. The sensory score in the treatment group (8.2 ± 1.4) was significantly better than that in the control group (5.2 ± 1.3) [32]. In another reported study involving 41 cases of fingertip skin defects, the patients were randomly assigned to the artificial dermis transplantation + rh-aFGF group (n = 21) and the artificial dermis transplantation group alone (n = 20). The results showed that the combination group achieved better outcomes in wound healing time, scar formation, skin color, and elasticity compared with the artificial dermis transplantation group alone [33].

### 2.7. Clinical Question 6: Combined Application with Wound Dressings

#### Recommendation 11 (Moderate): The Combined Application of rh-aFGF with Novel Dressings Facilitates the Healing of Chronic Wounds

Evidence and rationale: In a clinical study involving 80 cases of chronic wounds, the patients were randomly divided into two groups and treated with artificial dermis for repair. The overall response rate in the moist wound healing therapy + rh-aFGF was higher than that in the moist wound healing therapy group alone (100.00% vs. 85.00%). The wound healing time was significantly shorter (28.64 ± 5.11 days vs. 35.91 ± 6.08 days), and the wound scar score was lower in the combination group compared with the moist wound healing therapy group alone (3.10 ± 0.81 vs. 5.83 ± 1.14) [34]. The moist wound healing therapy involves debridement of a wound followed by coverage with a topical foam dressing, which is changed every 3 days until the wound is completely healed. This therapy maintains the wound in a moist environment and a hypoxic state locally through novel dressings, which can promote the formation of capillaries and the massive release of related immune factors and growth factors to reduce inflammatory responses [34]. Since a chronic wound develops in an alkaline environment [52], rh-aFGF can help to create an acidic microenvironment for it. Therefore, the wound microenvironment shifting from weakly alkaline to mildly acidic can effectively reduce wound inflammation and infection, facilitating wound healing [53,54]. Additionally, rh-aFGF is negatively charged in an acidic environment and can bind to cell membrane (positively charged) receptors to fully exert its biological activity and reduce the generation of inflammatory factors in a wound [1,15,55]. When combined with topical foam dressings, rh-aFGF can improve the repair effect in patients with chronic wounds. In another clinical study involving 96 cases of Wagner Grade II–IV diabetic foot ulcers, the patients were divided into the study group (49 cases) and the control group (47 cases). The study group received VSD + hydrocolloid silver sulfate dressing + irrigation with rh-aFGF solution + skin grafting, while the control group used VSD + hydrocolloid silver sulfate dressing + irrigation with normal saline + skin grafting. The results showed that both the hospital stay (20.16 ± 3.58 days vs. 22.72 ± 5.31 days) and wound healing time (16.18 ± 3.62 days vs. 18.79 ± 5.31 days) in the study group were significantly shorter than those in the control group. The healing rate in the study group was significantly higher than that in the control group (91.83% vs. 76.60%), and the scar scores at 3 and 6 months after treatment in the study group (4.18 ± 1.35 vs. 5.19 ± 1.33; 3.33 ± 1.08 vs. 4.19 ± 1.11) were significantly lower than those in the control group. These findings indicate that the multi-technique treatment regime combining VSD, hydrocolloid silver sulfate dressing, irrigation with rh-aFGF solution, and skin grafting can effectively shorten hospital stay and wound healing time, improve healing rates, and reduce scar formation in patients with diabetic foot ulcers [35].

### 2.8. Clinical Question 7: Management of Adverse Events and Precautions for Use of rh-aFGF

#### Recommendation 12 (Moderate): Use of rh-aFGF Rarely Causes Obvious Adverse Reactions. If Any Severe Adverse Reaction Occurs, rh-aFGF Should Be Discontinued Immediately. If Itching and Mild Rash Are Observed, Symptomatic Treatment Can Be Provided, and Its Use Can Be Continued Under Close Observation. If Symptoms Worsen, Discontinuation Is Recommended

Evidence and rationale: A clinical study involving 253 patients treated with rh-aFGF for chronic ulcerative wounds reported that two cases of itching and one case of mild rash occurred after use of rh-aFGF, while eight cases experienced mild itching, and three cases developed rashes in the control group receiving topical moist exposed burn ointment followed by spraying of normal saline [22]. In another randomized controlled clinical study involving 194 cases of refractory wounds treated with VSD + rh-aFGF, it was found that only 29.2% of the patients in the treatment group experienced wound paresthesia, compared with 86.5% in the control group; the wound infection rate in the treatment group was 8.1% versus 14.5% in the control group, indicating that application of rh-aFGF has a low incidence of adverse reactions and does not increase the risk of wound infection [30]. Multiple clinical studies on the combined use of rh-aFGF and VSD reported no severe adverse events [25,26,27,28,29,30]. For itching, oral antihistamines such as cetirizine or loratadine were administered; intramuscular diphenhydramine and intravenous dexamethasone were used for rashes, along with topical application of calamine lotion. As a result, the symptoms were effectively alleviated. Therefore, itching or mild rash after the application of rh-aFGF can generally be treated symptomatically. If symptoms worsen, discontinuation is recommended.

The following precautions should be observed during use to ensure the effectiveness of medication: ① Clean the skin before use. Directly spray or apply rh-aFGF onto the skin surface. Allow it to dry before using other agents. If protein denaturants (iodine, alcohol, hydrogen peroxide, heavy metals, etc.) are used, it is recommended to rinse the area with normal saline before using this product. ② Avoid contamination during the dissolution process and try to maintain a contamination-free environment during use. ③ For wounds at risk of infection, topical anti-infective agents can be combined. ④ It is not recommended for use in hypertrophic scars. ⑤ If any adverse reaction is observed, discontinue its use immediately. ⑥ Use with caution in athletes. ⑦ Before applying this product, if wound bed preparation involves protein denaturants such as iodine tincture, ethanol, or hydrogen peroxide, it is essential to thoroughly rinse the area with saline before administering rh-aFGF. This precaution is necessary to prevent any impairment of activities. From a study on full-thickness skin defects in a rabbit model, rh-aFGF at doses below 900 U/cm^2^ (10 times the clinically recommended dose) was observed to be safe and to effectively promote wound healing [56]. To date, there have been no reported cases of tumorigenesis associated with the local topical application of rh-aFGF on wounds.

## 3. Materials and Methods

This consensus has been registered on the Practice guideline REgistration for transPAREncy (PREPARE) (ID: PREPARE-2025CN265, http://www.guidelines-registry.org/, accessed on 30 December 2025), and the consensus protocol has been uploaded.

### 3.1. Consensus Writing Group and Its Responsibilities

The consensus writing group consists of more than 20 experts from multiple disciplines, including burn and plastic surgery, wound repair, orthopedics, and evidence-based medicine. It comprises advisors, a group leader, an expert panel, a methodology expert panel, a drafting panel, a clinical question consultation expert panel, an evidence evaluation panel, and a secretariat.

The main responsibilities of the advisors and group leader included inviting experts from multiple disciplines to form other panels, formulating the consensus writing schedule, determining the theme and scope of the consensus, approving the consensus protocol, managing declarations of conflicts of interest, providing methodological guidance and quality control, supervising the consensus development process, organizing consensus workshops, approving recommendations, approving the publication of the expert consensus, and assessing the need for future updates.

The main responsibilities of the expert panel included defining the scope of the expert consensus in terms of study subjects, interventions, comparison methods, and outcome measures based on PICO (P: patient, I: intervention, C: comparison, O: outcome); assessing the importance of clinical questions and outcome indicators; completing the Delphi expert questionnaires and participating in multiple discussions to agree on recommendations; completing the registration on PREPARE; conducting systematic and comprehensive searches of the literature and evidence synthesis; collecting and screening clinical questions and outcome measures and formulating clinical questions based on the PICO principle; designing the Delphi expert questionnaires, summarizing and statistically analyzing the questionnaire results, and providing feedback to the expert panel; and drafting the expert consensus and revising it based on the comments and suggestions from the expert panel. The clinical question consultation expert panel participated in questionnaire surveys and consensus discussions.

### 3.2. Search of the Literature and Evidence Evaluation

**Figure 3:**
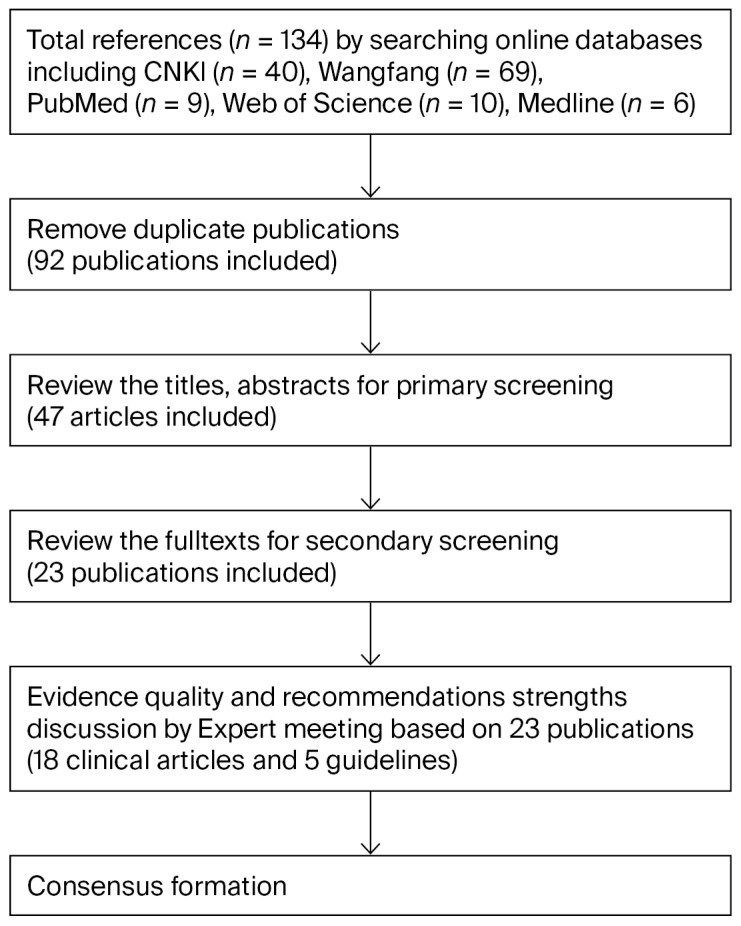
Diagram of the development of expert consensus on rh-aFGF for chronic refractory wound treatment.

The Chinese and English literature related to CRWs and rh-aFGF published up to 1 April 2025, was searched. Chinese search terms included chronic refractory wound, refractory, chronic wound, ulcer, diabetic foot, pressure ulcer, vascular ulcer, and decubitus. Chinese databases searched were China National Knowledge Infrastructure (CNKI) and Wanfang Data. English search terms included rh-aFGF, refractory wound, acid fibroblast growth factor, refractory, ulcer, diabetic foot, pressure sores, pressure ulcer wound, varicose ulcer, and decubitus. English databases searched were PubMed, Medline, and Web of Science. Priority was given to clinical application studies of rh-aFGF with indications for chronic wounds/chronic refractory wounds. The duplicate literature across databases was manually removed. Clinical application studies that did not use rh-aFGF were excluded. Non-controlled trials and studies using rh-aFGF in both the test and control groups were excluded. Case reports, the literature without available efficacy measures, non-clinical studies, and review articles were excluded; the literature screening process is illustrated in Figure 3.

The methodological quality (e.g., risk of bias) of the included randomized controlled trials was assessed using the Cochrane risk of bias tool. The screening and quality assessment of the above literature were independently completed by at least two members of the drafting panel. Two methodology experts reviewed and assessed the recommendations and their supporting evidence in each round and fed back comments to the drafting panel for further revision. Formal assessment of the quality of all evidence was conducted using the GRADE (grading of recommendations assessment, development and evaluation) system (Table 3) [57]. The evidence quality was classified into three levels: high, moderate, and low (including very low). The level of evidence quality can be downgraded due to the presence of risk of bias, inconsistency in study results, or publication bias and upgraded due to significant efficacy or the existence of an obvious dose–effect relationship.

### 3.3. Formation of Consensus Recommendations

The draft of recommendations and expert consensus was formulated by evaluating factors such as evidence quality, balance of benefits and harms, acceptability, and feasibility. The draft was discussed in meetings and revised repeatedly based on feedback from the expert panel. Two rounds of Delphi expert questionnaires were conducted to collect opinions on PICO-structured clinical questions [58]. Recommendations were finalized based on the evidence levels and the degree of expert consensus. The strength of consensus was graded according to the proportion of agreement. The standards for recommendation grade are more strict than the literature reports.

- Strong recommendation: Expert agreement ≥95%;
- Moderate recommendation: Expert agreement of <94%, ≥90%;
- Weak recommendation: Expert agreement of ≤89%, ≥85%;
- Items with an agreement <50% were not included.

## 4. Conclusions

rh-aFGF has been approved in China for more than two decades for wound healing treatment, including treatment of burns, ulcers, and surgical wounds. By reviewing previously published clinical studies on rh-aFGF in CRW and incorporating clinical experts’ experience, we have recommended its application for CRWs, which aims to standardize the application of rh-aFGF for CRWs. These recommendations serve as references only for clinical practitioners involved in wound repair and do not constitute legal grounds.

Given the relatively recent clinical introduction of rh-aFGF, further accumulation of clinical cases is required to clarify its therapeutic efficacy across various chronic non-healing wound types. Multi-center clinical studies, in particular, will provide robust evidence for future revisions and refinements to this consensus.

## Figures and Tables

**Table 1:** Growth factor products currently used for wound repair internationally.

Growth Factor	Dosage Form	Indication	Common Dose	Frequency	Approved Countries
aFGF	Powder	Burns, chronic refractory wounds	100 U/cm^2^	Once per day	China
bFGF	Powder/gel	Burns, chronic refractory wounds	150 U/cm^2^	Once or twice per day	China, Japan
EGF	Powder/gel	Chronic refractory wounds, surgical wound healing	400 U/cm^2^	Once per day	China, Cuba
PDGF	Powder/gel	Chronic refractory wounds	10 μg/cm^2^	Once per day	The United States, Europe
GM-CSF	Gel	Burns	1 μg/cm^2^, 10 μg/cm^2^	Once per day	China

**Table 2:** The characteristics and GRADE determination of rh-aFGF clinical studies.

Publications (First Author)	Indications	Sample Size	Intervention		Duration for Treatment	Clinical Outcomes	GRADE
			Experimental Group	Control Group			
Li, Y.T. (2023) [18]	chronic wound	40	rh-aFGF	/	30 days	Inhibiting inflammation; promoting wound healing; improving quality of life	Weak
Li, J. (2014) [19]	DFU, vascular ulcers, pressure ulcers	277	rh-aFGF	Moist exposed burn ointment	30 days	Promoting wound healing; reducing pain and exudate; accelerating granulation formation	Strong
Tan, Y. (2008) [20]	DFU	139	rh-aFGF	Rb-bFGF	6 weeks	Promoting wound healing; alleviating infection	Strong
Fu, L.Y. (2022) [21]	Pressure ulcers	30	rh-aFGF	Dressing	Until wound heals	Promoting wound healing	Moderate
Xu, J.S. (2018) [22]	DFU	99	rh-aFGF	Saline	60 days	Promoting wound healing; accelerating granulation formation	Strong
Sheng, X. (2024) [23]	Refractory diabetic foot	60	rh-aFGF + VSD	VSD	Until wound heals	Promoting wound healing; reducing pain and exudate; inhibiting inflammation	Strong
Chen, S.S. (2025) [24]	DFU	50	rh-aFGF + VSD	PRP	7 days	Promoting wound healing; inhibiting inflammation; alleviating infection	Moderate
Li, G. (2014) [25]	Refractory skin ulcer	60	rh-aFGF + VSD	dressing	21 days	Promoting granulation formation and wound healing; alleviating infection	Strong
Li, G. (2014) [26]	Refractory chronic wound	54	rh-aFGF + VSD	VSD + saline	10 days	Promoting wound healing	Moderate
Lin, Q.Y. (2015) [27]	Refractory skin ulcer	1200	rh-aFGF + VSD	VSD + saline	2 months	Promoting wound healing	Moderate
Shi, Y. (2017) [28]	Chronic osteomyelitis	153	rh-aFGF; rh-aFGF + VSD	Usual care	3 weeks	Promoting granulation formation and wound healing; alleviating infection	Strong
Wang, F.Y. (2015) [29]	Refractory chronic wound	80	rh-aFGF + VSD	VSD + saline	10 days	Promoting wound healing; enhancing sensory function	Moderate
Liu, T. (2015) [30]	Refractory chronic wound	194	rh-aFGF + VSD	VSD + saline	7 days	Promoting granulation formation and wound healing; decreasing scar formation	Strong
Liu, X. (2017) [31]	DFU	60	rh-aFGF + VSD	VSD + saline	7 days	Promoting wound healing; inhibiting inflammation; alleviating infection	Moderate
Liu, J.N. (2018) [32]	Soft tissue defects	42	rh-aFGF	Usual care	30 days	Promoting wound healing	Strong
Wu, L. (2023) [33]	Skin defects	41	rh-aFGF	Usual care	14 days	Promoting wound healing; decreasing scar formation; enhancing the sensory function	Moderate
Duang, J.J. (2024) [34]	Refractory chronic wound	80	rh-aFGF + Dressing	Dressing	4 weeks	Promoting wound healing; reducing pain and inflammation	Moderate
Pu, W.M. (2024) [35]	DFU	96	VSD + rh-aFGF + Dressing	VSD + dressing	7 days	Promoting wound healing; decreasing scar formation	Strong

**Table 3:** Evidence quality levels and descriptions based on the GRADE system.

Evidence Quality Level	Description
High	Very confident that the estimated effect is close to the true effect.
Moderate	Moderate confidence in the estimated effect; it is likely close to the true effect, but there remains a possibility that they are quite different.
Low	Limited confidence in the estimated effect; it may be substantially different from the true effect.

## Data Availability

The original contributions presented in this study are included in the article. Further inquiries can be directed at the corresponding authors.

## Abbreviations

The following abbreviations are used in this manuscript:

rh-aFGF	recombinant human acidic fibroblast growth factor
CRW	chronic refractory wound
bFGF	basic fibroblast growth factor
FGFR	fibroblast growth factor receptor
DFU	diabetic foot ulcer
VSD	vacuum sealing drainage
CNKI	China National Knowledge Infrastructure
EGF	epidermal growth factor
PDGF	platelet-derived growth factor
GM-CSF	granulocyte macrophage colony stimulating growth factor
GRADE	grading of recommendations assessment, development, and evaluation
